# Tumor microenvironmental modification by the current target therapy for head and neck squamous cell carcinoma

**DOI:** 10.1186/s13046-023-02691-4

**Published:** 2023-05-05

**Authors:** Kohei Okuyama, Tomofumi Naruse, Souichi Yanamoto

**Affiliations:** 1 Department of Periodontics and Oral Medicine, University of Michigan, 1600 Huron Parkway, Ann Arbor, MI 48105 USA; 2 University of Michigan Rogel Cancer Center, Ann Arbor, MI USA; 3 Department of Oral and Maxillofacial Surgical Oncology, Graduate School of Medical and Dental Sciences, Tokyo Medical and Dental University, Tokyo, Japan; 4 Department of Clinical Oral Oncology, Nagasaki University Graduate School of Biomedical Sciences, Nagasaki, Japan; 5 Department of Oral Oncology, Graduate School of Biomedical and Health Sciences, Hiroshima University, Hiroshima, Japan

**Keywords:** Head and neck squamous cell carcinoma, Immune checkpoint inhibitors, Cetuximab, Nivolumab, PD-1, CTLA-4, Tumor microenvironment

## Abstract

Current clinical and observational evidence supports the EXTREME regimen as one of the standards of care for patients with recurrent or metastatic head and neck squamous cell carcinoma (HNSCC) followed by the administration of immune checkpoint inhibitors (ICIs). In addition to the inhibition of the epidermal growth factor receptor (EGFR) pathway, cetuximab-mediated EGFR blockade has been shown to modulate tumor microenvironment (TME) characteristics, such as antibody-dependent cellular cytotoxicity (ADCC) activity, cytotoxic T-lymphocyte (CTL) infiltration into the tumor, anti-angiogenesis activity, and cytokine secretion via associated natural killer (NK) cells, etc.. On the other hand, there are reports that nivolumab affects the TME via Programmed cell death 1 (PD-1) inhibition, Interleukin-10 upregulation via T-cells, myeloid-derived suppressor cell-mediated immune escape induction, and tumor vessel perfusion by promoting CD8 + T-cell accumulation and Interferon-γ production in treatment-sensitive tumor cells. Actually, nivolumab administration can give T cells in the TME both immune superiority and inferiority. HNSCC treatment using cetuximab increases the frequency of FoxP3 + intratumoral effector regulatory T cells (Tregs) expressing CTL associated antigen (CTLA)-4, and targeting CTLA-4 + Tregs using ipilimumab restores the cytolytic function of NK cells, which mediate ADCC activity. Treg-mediated immune suppression also contributes to clinical response to cetuximab treatment, suggesting the possibility of the addition of ipilimumab or the use of other Treg ablation strategies to promote antitumor immunity. Moreover, also in hyper progression disease (HPD), intratumoral frequency of FoxP3 + effector Tregs expressing CTLA-4 is increased. Therefore, combination treatment with cetuximab plus anti-CTLA-4 antibody ipilimumab for HNSCC and this combination therapy after nivolumab administration for HPD may be expected to result in a higher tumor-control response. Based on the above evidence, we here suggest the efficacy of using these therapeutic strategies for patients with local-advanced, recurrent, and metastatic HNSCC and patients who do not respond well to nivolumab administration.

## Introduction

Until recently, patients with platinum-refractory recurrent or metastatic head and neck squamous cell carcinoma (R/M HNSCC) had poor prognoses and limited options besides the therapy including cetuximab [1]. Current field-based clinical and observational evidence supports the EXTREME regimen as the standard of care for fit patients with R/M HNSCC, followed by a new treatment option involving immune-checkpoint inhibitors (ICIs). Cetuximab targets epidermal growth factor receptor (EGFR) and interrupt oncogene signaling in tumors that have become oncogene-addicted. Moreover, it can result in the induction of innate and adaptive immune responses and the downregulation of immunosuppressive mechanisms [2–5]. It has also been observed that cetuximab-mediated EGFR blockade downregulates interferon-gamma (IFN-γ)-induced programmed death ligand 1 (PD-L1) expression in HNSCC; this may signify the restoration of the antitumor immune response [6, 7]. Cetuximab also drives the antibody-dependent cellular cytotoxicity (ADCC) of natural killer (NK) cells as well as the maturation and the crosstalk between NK cells and dendritic cells (DC). In contrast, it promotes the multiplication of immunosuppressive regulatory T cells (Tregs) in the tumor microenvironment (TME) [4]. Cetuximab-activated NK cells also secrete cytokines, which enhance antigen presentation [8]. In these respects, patients treated long-term with cetuximab may be under the multiplication of both positive (NK, dendritic cell) and suppressive cell types (Tregs, myeloid-derived suppressor cells (MDSCs)). Thus, the response to ICI treatment is limited and controversial [4, 8, 9].

The CheckMate-141 trial, a phase III trial aimed at investigating the suitability of nivolumab versus the investigator's choice of therapy for patients with R/M HNSCC, who had experienced tumor progression or recurrence within 6 months of platinum-based chemotherapy in the locally advanced R/M setting. Patient randomization was stratified based on prior cetuximab exposure to minimize imbalances in the treatment arms due to the reported immunomodulatory effects of cetuximab. Thus, the primary analysis showed that compared with the investigator's choice of therapy, nivolumab significantly improved survival in the overall study population and showed a potential advantage for patients without prior cetuximab exposure [10]. Since the discontinuation of cetuximab or nivolumab is usually due to uncontrolled disease progression (here, we do not consider the occurrence of severe adverse events as an exception), there is often a need to replace cetuximab with nivolumab, and thereafter, it may be considered to rechallenge with cetuximab in some cases. Under such conditions, some patients with R/M HNSCC show prominent tumor-suppressing effects owing to cetuximab administration following nivolumab administration and some previous studies have reported the excellent antitumor effect of cetuximab after nivolumab treatment [11–13]. There is also evidence that nivolumab recruits tumor-infiltrating lymphocytes, including CD4 + and CD8 + T cells, and upregulates IFN-γ-related chemokines [14]. However, detailed investigations of the TME are not enough to explain the biological states of tumors.

In this narrative review, we summarize and discuss background evidence regarding the biological effects of nivolumab after cetuximab administration and of cetuximab after nivolumab administration and suggest how the efficacy of the regimens can be optimized for patients with HNSCC who do not show a good response to these current targeted therapies.

### Biological contribution of nivolumab other than PD-1 inhibition

#### Current general understanding of ICI strategy

The therapeutic activity of ICIs results from a complex interplay among intrinsic cancer cell traits, the TME, and the host immune system [15]. With the integration of next-generation sequencing in clinical practice for tumor molecular profiling for personalized cancer treatment, a rise in somatic alterations that could influence response to immunotherapy has been observed [16]. Somatic mutations in mismatch repair genes and high microsatellite instability lead to a particular immunophenotype characterized by increased responsiveness to ICIs [17]. Furthermore, increasing tumor mutation burden, usually defined as the number of nonsynonymous mutations per megabase of sequenced DNA, is also a predictive biomarker for better response to programmed cell death 1 (PD-1) blockade and improved clinical outcomes [18, 19]. It is also worth noting that cancer cells are capable of increasing PD-L1 expression in response to a robust immune attack that is usually mounted by tumor antigen-specific T cells, and this process is largely dependent on effective immune recognition, which in turn, is dependent on increased somatic mutation and neoantigen burden [20]. In addition, EGFR-driven tumors have been reported to possess a lower mutational burden [21].

#### IFN- γ and anti-PD-1 therapy

Other than PD-1 inhibition, studies on the biological dynamics of nivolumab in HNSCC are still limited. Indeed, even though nivolumab mainly targets the blockade of the formation of the PD-1/PD-L1 axis, the surrounding effector (i.e., cytokines, chemoattractants, tissue acidosis, etc.) dynamics might also be affected by this blockade, and may modulate their population in the TME. Observations corresponding to patients who are responsive to ICI suggest that blocking PD-1 increases the number and function of CD8 + T cells infiltrating the TME [22]. From another point of view, ICIs increase tumor vessel perfusion by promoting CD8 + T-cell accumulation and IFN-γ production in treatment-sensitive breast and colon cancer cell lines, but not in treatment-resistant models [23, 24]. Moreover, Ding et al. reported that combination therapy with nivolumab and IFN-γ shows a synergistic effect on PD-1 blockade compared with IFN-γ or nivolumab alone in pancreatic cancer [25]. These pieces of evidence emphasize the positive antitumor role of IFN, even in HNSCC. Furthermore, a stimulator of IFN genes (STING), has been shown to exhibit antitumor activity. STING is frequently inhibited in the TME, and this contributes to the escape of cancer cells from innate immune sensing. It is also expressed in endothelial cell vasculatures, suggesting that combining STING agonists with anti-PD-1 or anti-PD-L1 antibodies and anti-angiogenic agents could overcome primary or secondary resistance to ICI [26]. Thus, TME modulation after nivolumab administration is promising from the perspective of IFNs and STING dynamics. However, the prolonged, over-activation of STING may also induce negative situations for antitumor immunity (reviewed in [27]). Whereas STING facilitates antitumor immune response by promoting the infiltration of effector cells and eradication of tumor cells, constant STING activation may hamper immune response by inducing the infiltration of immune suppressive cells, such as Treg and MDSC, and upregulating the expression of PD-L1 on tumor cells and PD-1 on T cells. Moreover, aberrant STING activation directly inhibits T cell proliferation and even promotes apoptosis of lymphocytes. To achieve the maximum antitumor effect using the STING agonist, it is likely that the desired STING activation state must be maintained during the desired time period of administration.

#### MDSCs and anti-PD-1 therapy

Another strategy by which the tumor- and immune-microenvironment can be modified using nivolumab is by interfering with MDSC function. Specifically, MDSCs exert their immunomodulatory effects via diverse mechanisms, including Arginase-1-mediated depletion of l-arginine and nitric oxide (NO) production via NO synthase 2 (NOS2). It is reported that Arginase-1 starves L-arginine in the TME; thus, it limits T-cell proliferation [28]. In addition to L-arginine depletion, NO production transforms the TME to promote immune escape [29]. It has also been observed that short-term NO exposure reversibly inhibits T cells, while prolonged exposure leads to T cell apoptosis [30]. This MDSC-related driven-immune escape cascade may contribute to developing the hyper progressive disease (HPD), implying that the appropriate control of MDSCs may improve the response rate of ICI treatment for such disease. Moreover, using pancreatic cancer cells, Thakur et al. reported that reduced MDSC accumulation is accompanied by significantly lower levels of COX2 and PGE2, and their downstream effector molecule, Arginase-1. It is also associated with significantly higher levels of tumor necrosis factor (TNF)-α, Interleukin (IL)-12, and the chemokines, CCL3, CCL4, CCL5, CXCL9, and CXCL10 in antibody-armed activated T-cells, thus, it brings about a Th1 cytokine-enriched microenvironment. Some of the chemokines are also STING downstream effector cytokines and relate to the activation of STING/type I IFN signaling pathway. This activation then facilitates CD8 + T cells to infiltrate into the tumor. The results from their study indicated that antibody-armed activated T-cells can suppress MDSC differentiation and attenuate their suppressive activity via the downregulation of COX2, PGE2, and Arginase-1 pathways, which are potentiated in the presence of Th1 cytokines, suggesting that Th1 enriching immunotherapy may be beneficial in pancreatic cancer treatment [31]. Thus, MDSCs play important roles in cancer cell metabolism and TME dynamics, and their control might increase therapeutic potential with respect to the targeting of the tumor immune microenvironment.

Furthermore, immunogenic cell death is characterized by the release of danger-associated molecular patterns (DAMPs) by dying cells, activation of antigen-presenting cells (APCs) upon DAMP binding to specific receptors, tumor neoantigen uptake, subsequent activation of cytotoxic T lymphocyte (CTL)-based immune response, and the establishment of an immune memory, which eliminates tumor cells [32]. It is also known that the secretion of type I IFNs from these cells acts as a DAMP and results in the production of CXCL10 chemokine which acts as a chemoattractant for CTL [33]. This immune modulation can also drive antitumor immunity.

#### Other biological modifications by anti-PD-1 therapy

**Fig. 1:**
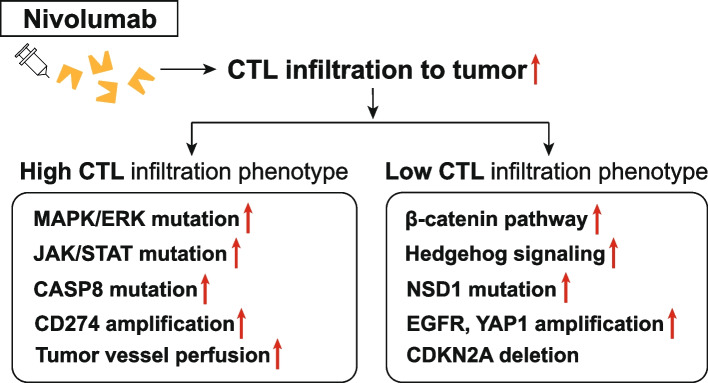
Differences in the responses of high- and low-CTL tumors to nivolumab administration

Yue et al. reported that BRAF and/or MEK inhibition on nivolumab-induced T cell activation is due to alterations in the activation of AKT and T cell receptor (TCR) signaling pathways. They investigated the combinatorial effects of mitogen-activated protein kinase (MAPK) pathway inhibitors on nivolumab-induced T cell responses by assessing cytokine production, the expression of T cell proliferation and activation markers, and functional markers, such as Granzyme B, as well as the activation of various signaling pathways in T cells. However, they concluded that combination therapy using nivolumab with either a BRAF inhibitor, a MEK inhibitor, or both might have a limited synergistic effect on nivolumab-induced T-cell activation. They further stated that these combination therapies might not be of benefit to most patients who are receiving ICI treatment [34]. In addition, Saloura et al. reported that, while HNSCC, with a low CD8 + T cell-inflamed phenotype, shows enriched β-catenin and Hedgehog pathways, NSD1 mutations, and EGFR and YAP1 amplifications, a high CD8 + T cell-inflamed phenotype was found to be associated with MAPK/extracellular signal-regulated kinases (ERK) and Janus kinase (JAK)/signal transducer and activator of transcription (STAT) pathways, CASP8 mutations, and CD274 amplifications (Fig. 1) [35]. Because of the complex confounding differences in gene expression, signaling, and activated enzymes in the TME, therefore, the number of T lymphocytes and the inflammation intensity in the tumor cannot simply predict the ICI treatment outcomes, and sticking to one biomarker or one therapy may result in a treatment failure.

Other, Lamichhane et al. observed elevated IL-10 levels induced by anti-PD-1 antibody treatment in vivo [36]. Moreover, Harper et al. reported that nivolumab induces potent IL-10 secretion in T cells by activating the MAPK pathway [37], in which T cells can then activate their growth and metabolism, possibly leading to T-cell immune superiority. Conversely, in an in vitro study, Puntigam et al. reported that nivolumab treatment significantly reduced the level of the receptor, PD-1 in all analyzed T-cell populations. Their data suggested that IL-10 may confer a heterogeneous T-cell response to nivolumab [28]. It is necessary to investigate whether the interesting in vitro result can be reproduced in vivo to further clarify the mechanisms underlying the modulated antitumor immunity observed after PD-1 blockade. Actually, IL-10 is one of the most important immunoregulatory cytokines that regulate T-cell responses by modulating multiple signaling pathways [36–39]. The engagement of IL-10 with its receptor also activates and expands multiple signaling pathways, particularly the JAK-STAT3 pathway [40–42].

**Fig. 2:**
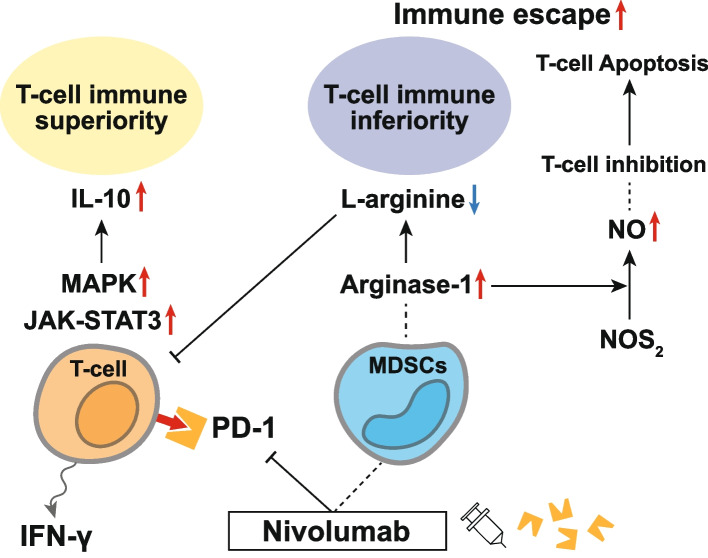
Immune superiority and inferiority of T-cells in the TME following nivolumab administration. IL-10 is one of the most important immunoregulatory cytokines that regulate T-cell responses by modulating multiple signaling pathways. Nivolumab induces potent IL-10 secretion in T cells via the activation of the MAPK pathway, in which T cells can activate T cell growth and metabolism, possibly leading to T-cell immune superiority. Based on an in vitro study, nivolumab treatment significantly reduces PD-1 levels in all T-cell populations, suggesting that IL-10 may confer a heterogeneous T-cell response to nivolumab. The engagement of IL-10 with its receptor also activates multiple signaling pathways, particularly the JAK-STAT3 pathway, indicating that nivolumab triggers several biological pathways in the TME. MDSCs exert their immunomodulatory effects via diverse mechanisms, including Arginase-1-mediated depletion of l-arginine and NO production via NOS2. Arginase-1 starves L-arginine in the TME, thus limiting T-cell proliferation. In addition to L-arginine depletion, NO production transforms the TME to promote immune evasion and prolonged NO exposure leads to T-cell apoptosis, causing T-cells to show TME immune inferiority

Taken together, nivolumab administration can give T cells in the TME both immune superiority and inferiority (Fig. 2). Considering a heterogeneous T-cell response to anti-PD-1 antibody treatment, it might be necessary to focus on a more individualized TME status and identify promising prognostic factors to arrange the order-made therapy targeting immunoregulatory cytokines. Such treatment strategies that involve adjunctive sensitization with anti-tumor drugs will be a future highlight, especially in immunotherapy.

### Biological contribution of cetuximab other than EGFR blockade

#### EGFR pathway inhibition and immunomodulation

EGFR-activating mutations contribute to an immunosuppressive TME and patients with EGFR mutations may not respond favorably to anti-PD-1 or anti-PD-L1 therapy [19]. Epidemiological studies have suggested the existence of an inverse relationship between oncogenic EGFR mutations and PD-L1 expression [43]. Furthermore, meta-analyses of multiple immunotherapy trials compared to standard chemotherapy demonstrated that only EGFR-wild-type patients benefit from anti-PD-1 or anti-PD-L1 antibodies, while patients with EGFR-mutated tumors did not achieve improved overall survival or longer progression-free survival in patients with advanced non-small cell lung carcinoma (NSCLC) while on ICI therapy [44]. Moreover, based on a large cohort of head and neck cancer specimens, Concha-Benavente et al. reported that the overexpression of wild-type EGFR is significantly correlated with JAK2 and PD-L1 expression. They further showed that PD-L1 expression is induced in an EGFR- and JAK2/STAT1-dependent manner. Specifically, they reported that JAK2 inhibition prevents PD-L1 upregulation in tumor cells and enhances their immunogenicity [7]. Thus, to benefit from anti-PD-1 antibody therapy, evaluating EGFR status (high/low and wild-type/mutated) combined with PD-1 expression before the administration may be a more reliable biomarker.

In addition, another potential immunomodulator is IL-6, a tumor-suppressive cytokine that promotes tumor cell proliferation and survival in the TME [45]. Cetuximab repolarized tumor-associated macrophages (TAMs) from M2-like to M1-like phenotypes, mainly by suppressing the IL-6 expression through NF-κB and STAT3 pathways [46]. IL-6 stimulates the phosphorylation of STAT3 through JAK 1 and 2. Moreover, DC maturation is suppressed by tumoral secretion of STAT3-induced cytokines, in particular IL-6 [47]. A preclinical JAK 1/2 inhibitor, AZD1480, abrogated IL-6-induced STAT3 phosphorylation and suppressed the growth of human solid tumor xenografts with constitutive STAT3 activity [48]. Taken together, it is necessary to understand how this immunomodulation in the TME provided by cetuximab affects subsequent ICI treatment outcomes and further investigation will be required to establish the most effective strategy for an individual patient with HNSCC.

#### Anti-angiogenesis of cetuximab and its contribution to subsequent treatment

Cetuximab treatment typically results in reduced angiogenesis or tumor vascularization. In general, anti-angiogenic normalization is mediated by vessel pruning, which reduces interstitial pressure, increases pericyte coverage, and restores intratumoral perfusion [49, 50]. Reportedly, these changes improve tumor drug and oxygen penetration, thereby enhancing chemotherapy and/or radiotherapy outcomes [51]. In the first detailed in vivo and in vitro study in this regard by Huang et al., the treatment of HNSCC with the anti-EGFR antibody, C225 reduced cell-to-cell interaction between human umbilical vascular endothelial cells, resulting in the disruption of tube formation. The effect of C225 was then further examined using an in vivo tumor xenograft neovascularization model of angiogenesis. The results revealed that systemic treatment with C225 not only reduced tumor growth and the number of blood capillaries but also hindered the growth of established vessels toward the tumor. These results provide evidence that treatment with anti-EGFR antibodies may suppress tumor-induced neovascularization and metastasis in HNSCC [52]. In a human HNSCC tissue microarray, increased EGFR expression was found to be correlated with increased hypoxia-inducible factor (HIF)-1α and microvessel density. Luwor et al. previously demonstrated that the inhibition of vascular endothelial growth factor (VEGF) by cetuximab occurs at the level of transcription in response to a reduced level of HIF-1α. This observation was further confirmed by testing therapeutic strategies that combine cetuximab with approaches that inhibit the function of VEGF or the VEGF receptor [53]. Prince et al. reported adjuvant anti-angiogenic therapy, utilizing an anti-VEGFR2 and anti-VEGFR3 antibody (co-administration) as a novel therapeutic method for enhancing cetuximab uptake compared with the control. These agents restore intratumoral fluid dynamics and improve drug perfusion [54]. Treatment for enhancing the anti-angiogenesis effect of cetuximab is still under development.

**Fig. 3:**
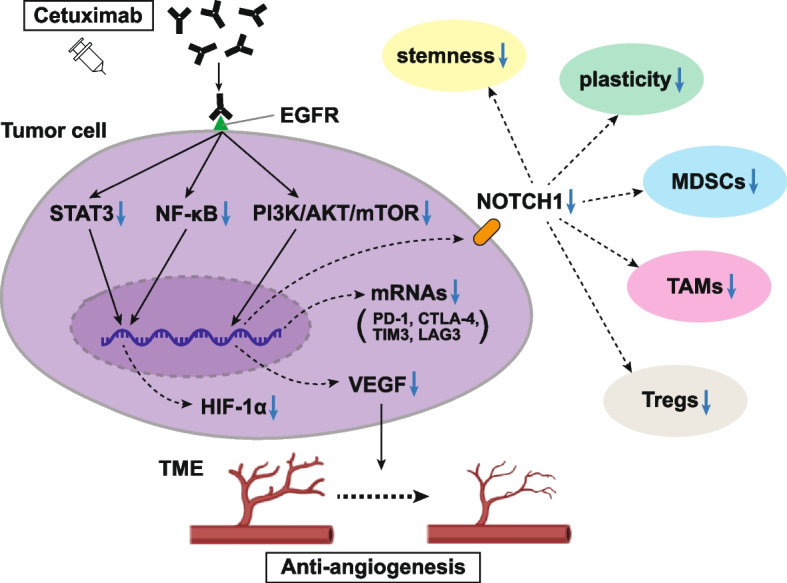
Neovascularization suppression and TME modulation by cetuximab. Treatment with cetuximab suppresses tumor-induced neovascularization in HNSCC. Increased EGFR expression shows a correlation with increased HIF-1α level and microvessel density. The previously demonstrated inhibition of VEGF by cetuximab occurs at the transcriptional level in response to reduced levels of HIF-1α. Moreover, cetuximab has been shown to attenuate the secretion of proangiogenic factors in tumor cells, such as VEGF and IL-8. Cetuximab inhibits tumor-induced angiogenesis by downregulating HIF-1α and Notch1, resulting in reduced angiogenesis and tumor shrinkage. The Notch signaling pathway plays an important role in blood vessel formation and remodeling. The inhibition of Notch signaling also reduces the number of MDSCs, TAMs, and Tregs within the tumor and inhibits the mRNA and target protein expression of the most relevant immune checkpoint molecules: PD-1, CTLA4, TIM3, and LAG3

Accumulated evidence has revealed that anti-angiogenic agents induce other signaling modulations in TME-related anti-angiogenesis. Wang et al. showed that cetuximab inhibits tumor-induced angiogenesis in vitro and in vivo by significantly downregulating HIF-1α and Notch1, resulting in reduced angiogenesis and tumor shrinkage [55]. Actually, Troy et al. reported that Notch signaling plays an important role in blood vessel formation and remodeling [56]. Some of the most intensely investigated Notch signaling pathway-related phenotypes observed in cancers are closely related to generating and modulating TME and intra-tumor heterogeneity. It was also reported that high expression levels of *NOTCH1* mRNA in the tumor tissues correlate with improved patient outcomes and longer survival [57], which seemed to contribute to a tumor-suppressor-like function of the Notch pathway, which is lost by alterations of this. Moreover, Kałafut et al. reported that the stemness and plasticity of HNSCC cells are strongly promoted by Notch signaling [58]. Mao et al. showed that the inhibition of the Notch signaling pathway is associated with reduced MDSCs, TAMs, and Tregs within emerging mouse tumor tissues, whereas the upregulation of the Notch1 downstream target, HES1, is significantly correlated with increased MDSCs, TAMs, and Tregs. In addition, the inhibition of Notch signaling significantly inhibits the mRNA and protein expression levels of the most relevant immune checkpoint molecules, PD-1, cytotoxic T lymphocyte-associated antigen-4 (CTLA-4), T-cell immunoglobulin and mucin-domain containing-3 (TIM-3), and lymphocyte-activation gene-3 (LAG-3), all of which represent targets for approved or developmental ICIs (Fig. 3) [59]. The expression of CTLA-4 [60], TIM-3 [61], and LAG-3 [62] immune checkpoints are typically upregulated in dysfunctional and/or exhausted T-cells. Therefore, the inhibition of the Notch signaling pathway can largely modify TME and further research will be required to confirm whether this Notch inhibition by cetuximab can contribute to sensitizing subsequent ICI treatment.

### Biological dynamics of cetuximab FOLLOWED BY anti-PD-1 antibody therapy 

#### Indications from clinical trials 

The approval of pembrolizumab and nivolumab for patients with R/M HNSCC who progressed on or after platinum-containing treatment was based on the results from the KEYNOTE-012 [63] and CheckMate 141 [64] trials. Ferris et al. reported that in the CheckMate 141 trial, nivolumab appeared to improve efficacy compared to the investigator's treatment choice, regardless of prior cetuximab use, supporting its use in patients with R/M HNSCC with or without prior cetuximab exposure. Importantly, the decrease in the risk of death associated with nivolumab administration compared with that associated with the investigator's treatment choice was greater for patients without prior cetuximab exposure (OS, 8.2 months; HR, 0.52; 95% CI, 0.35–0.77) than for those with prior cetuximab exposure (OS, 7.1 months; HR, 0.84; 95% CI, 0.62–1.15) [64]. The timing of cetuximab treatment is important because, as summarized above, at first glance, it appears that cetuximab can modulate the TME to provide a suitable stage for subsequent ICI treatment. However, the abovementioned clinical trial did not indicate this. Whereas cetuximab has been shown to significantly downregulate IFNγ-induced PD-L1 expression in head and neck tumor cell lines [7], tumor PD-L1 expression (< 1% versus ≥ 1%) was similar in patients with and without prior cetuximab exposure in CheckMate 141 trial [64], indicating that differences in response to nivolumab between these patient groups are not related to the effect of cetuximab on tumor PD-L1 expression.

#### ADCC activities of cetuximab and interaction with anti-PD-1 therapy

**Fig. 4:**
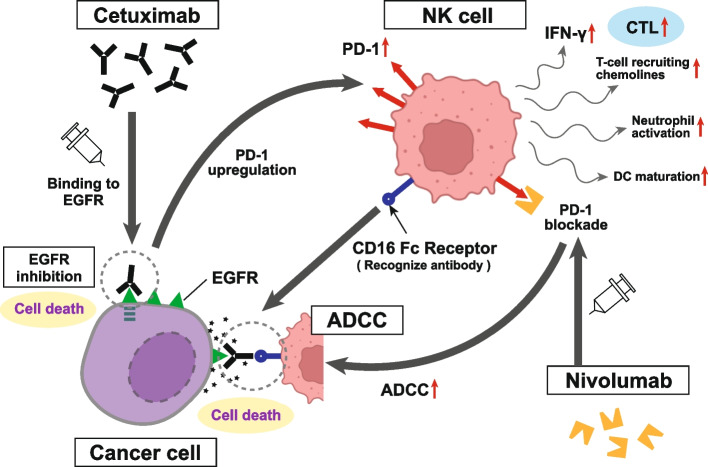
ADCC activity of cetuximab treatment and the effect on nivolumab administration. Pre-clinical studies have demonstrated the ability of cetuximab to stimulate ADCC and affect antitumor immunity. In vitro, cetuximab can mobilize NK cells, activate neutrophils, and stimulate DC maturation. This contributes to an extra antitumor effect in addition to simple EGFR inhibition. NK cell activation then produces IFN-γ, which promotes PD-L1 expression in tumor cells and emits T cell-recruiting chemokines that activate a high density of CTL in the TME. Cetuximab treatment also upregulates PD-1 expression in NK cells and maximizes antitumor effects. Additionally, PD-1 blockade increases cetuximab-mediated ADCC against PD-L1-high HNSCC cells without EGFR amplification, indicating that the combination of anti-EGFR antibodies with ICIs can enhance both innate and acquired antitumor immune responses

Preclinical studies have demonstrated the ability of cetuximab to stimulate ADCC and affect antitumor immunity. In particular, in vitro evidence shows that cetuximab can mobilize NK cells, activate neutrophils, and stimulate DC maturation [65–68]. Furthermore, enhanced cytotoxic activity has been documented based on ex vivo ADCC assays involving patients with R/M HNSCC receiving cetuximab-based therapy, and specifically, induced ADCC was found to be associated with positive clinical outcomes [65]. It has also been proposed that ADCC stimulation is an underlying mechanism for the clinically meaningful activity of cetuximab and the comparatively notable response rates observed during first- and second-line treatments in patients with R/M HNSCC [69]. This is responsible for the extra antitumor effect other than simple EGFR inhibition. In addition, cetuximab treatment upregulates PD-1 expression in NK cells to maximize antitumor effects. On the other hand, it has also been observed that PD-1 blockade enhances cetuximab-mediated ADCC against PD-L1-high HNSCC cells without EGFR amplification (Fig. 4). In this regard, Concha-Benavente et al. investigated that combining anti-EGFR antibodies with an anti-PD-1 inhibitor could enhance both innate and acquired antitumor immune responses against EGFR-amplified [70]. In clinical practice, phase II clinical trials involving patients with R/M HNSCC revealed that pembrolizumab in combination with cetuximab shows promising clinical activity for R/M HNSCC (NCT03082534) [71]. Highlighting ADCC activity, a phase I trial performed focusing TIGIT (T-cell immunoreceptor with immunoglobulin and immunoreceptor tyrosine-based inhibitory motif domain), which is a co-inhibitory receptor of T-cell and NK cell activity, revealed that etigilimab (TIGIT inhibitor) has an acceptable safety profile and shows preliminary evidence of clinical benefits when administered alone or in combination with nivolumab [72]. Thus, it is expected to be a better course for further investigation in clinical trials. In the preclinical HNSCC model, Patin et al. also reported that increased TIGIT expression on NK cells based on the inhibition of TIGIT signaling represents an effective treatment strategy to boost NK-cell activity and ADCC [73].

#### Antibody-dependent cellular phagocytosis (ADCP) and macrophages

Liu et al. reported that elevated PD-L1 levels in macrophages, in both tumor and stromal compartments, are correlated with high PD-L1 levels in tumors, as well as high CD8 and CD68 levels. Moreover, a high PD-L1 expression level in macrophages shows a correlation with better overall survival, while a high PD-L1 expression level in tumor cells shows opposite results [74]. Importantly, most clinically used anticancer monoclonal antibodies are of the IgG isotype, which can eliminate tumor cells through NK cell-mediated ADCC and macrophage-mediated ADCP. IgG isotype, however, ineffectively recruits neutrophils as effector cells [75]. Su et al. reported that after ADCP, macrophages inhibit NK cell-mediated ADCC and T cell-mediated cytotoxicity in breast cancers and lymphomas. They showed the recruitment of absent in melanoma 2 (AIM2), a DNA-sensing protein for the activation of the caspase-1 inflammasome, to the phagosomes following ADCP and activated by sensing the phagocytosed tumor DNAs, subsequently upregulating PD-L1 and indoleamine 2, 3-dioxygenase and causing immunosuppression in TME [76]. These varied mechanisms ultimately result in more efficient activated tumor-specific T cell apoptosis and a decreased efficacy of T effector cell-mediated tumor cell apoptosis.

#### Tumor immune status after cetuximab treatment

Several studies have shown that inhibiting EGFR using anti-EGFR inhibitors modulates the tumor immune microenvironment, with the effects including the enhancement of MHC class I and II expression, a decrease in the suppressive function of Tregs, the promotion of CTL activity, and reduced T cell apoptosis [77–80]. Anti-EGFR inhibitor-induced upregulation of MHC class I/II expression as well as the inactivation of GSK-3β/FoxP3 dampens Treg function and increases CTL activity is known as the main event that explains the modulation of the tumor immune microenvironment by cetuximab. Many tumors downregulate MHC expression to evade detection by the immune system. Particularly, in NSCLC, the expression level of PD-L1 is reduced by EGFR inhibitors [81–83]; however, it is upregulated in some patients with acquired resistance to EGFR inhibitors [84–86]. Bhola et al. generated HNSCC models of acquired cetuximab resistance by isolating clones from HNSCC cell lines grown in a culture medium containing increasing concentrations of cetuximab and showing higher PD-L1 expression levels than their isogenic parental controls [87]. Moreover, we previously summarized the mechanisms in which cetuximab plays a potential role in the synthesis of PD-L1 via the inhibition of its ubiquitination in HNSCC [88]. Elucidating the mechanism for modulating antitumor immunity, such as PD-L1 upregulation via the ERK, AKT-mechanistic target of rapamycin (mTOR), and STAT3 pathways, should be needed. This investigation may be going to explain the biological efficacy of cetuximab followed by ICI treatment. Importantly, Kansy et al. investigated the TCR richness and clonality in samples pre- and post-treatment in a prospective clinical trial of neoadjuvant cetuximab for HNSCC and revealed that neoadjuvant cetuximab treatment significantly increased the number of unique TCR sequences in peripheral blood mononuclear cells, which was more prominent in the clinical responder patients compared to non-responders. Moreover, a trend toward TCR gene focusing was observed in TILs in post-treatment samples. These data showed an influence of both peripheral quantity and intratumoral quality on adaptive immunity in cetuximab-treated patients [89]. Taken together, cetuximab treatment does not provide a negative factor that biologically reduces the effectiveness of subsequent ICI treatment.

#### Contribution of Extracellular matrix (ECM) to the treatment response of cetuximab and nivolumab

So far, there have demonstrated a lot of studies about cancer-associated fibroblasts (CAFs) as the main producers of ECM, which is directly involved in drug resistance mechanisms [90, 91], TME modulation [92, 93], ECM remodeling [94], and tumor aggressive behavior [95]. Galindo-Pumariño et al. generated an ECM model by using normal fibroblast, CAFs, and cell lines (fibroblast and tumor cells) and identified its role in the cetuximab-resistance processes of colorectal cancer (CRC) cells mediated by SNAI1-expressing fibroblasts in vitro and also showed the matrices generated by Snai1-knockout mouse embryonic fibroblasts (MEF) confer less resistance on cetuximab than wild-type MEF-derived matrices in vivo. They suggested the possible use of SNAI1 expression in CAFs as a predictive biomarker of response to cetuximab treatments in patients with CRC [96]. Moreover, in head and neck cancer, Prieto-Fernández et al. highlighted the drugs, specifically targeting EGF, Insulin-like growth factor, and Platelet-derived growth factor signaling pathways, emerge as excellent strategies to block functionally CAF-enhanced stemness and tumor-sphere forming ability, to consequently reduce the stemness of cancer [94].

ECM, especially CAFs, have emerged as essential factors in the modulation of the immune system, supporting the generation of an immunosuppressive environment [97]. Studies are showing that a high abundance of CAFs correlates with immune exclusion and ICI failure [98], while others reported that the absence of CAFs is associated with lower immune infiltration [99]. This discrepancy might be explained by the different observations in CAF's behavior that could be due to differences in their activation status and the presence of distinct CAF subpopulations coexisting in the TME as well as their interaction with the rest of the tumor-stroma components. As one example, Obradovic et al. reported the existence of different CAF subpopulations and demonstrated their functional importance in modulating the immunoregulatory milieu of cancer. They identified some CAFs subtypes as useful biomarkers to predict resistance to nivolumab. These identified actionable CAF subtypes can be used as a biomarker for treatment response and resistance [100]. Moreover, the ECM stiffness developed by CAFs is one of the important factors in the response of nivolumab: the generation of fibrosis, characterized by a strong cross-linked ECM acting as a physical barrier, which impairs immune cell infiltration thereby facilitating immune escape [101]. This ECM stiffness could also be considered a potential therapeutic target to increase tissue permeability, and consequently, improve immune cell penetration, ultimately leading to cancer cell death [94].

Taken together, it is possible to predict the therapeutic effect of cetuximab and nivolumab partly based on the status of the ECM especially focusing on CAFs. It is important to select the effective treatment individually based on the evidence.

### Biological dynamics of cetuximab FOLLOWING anti-PD-1 antibody treatment 

**Fig. 5:**
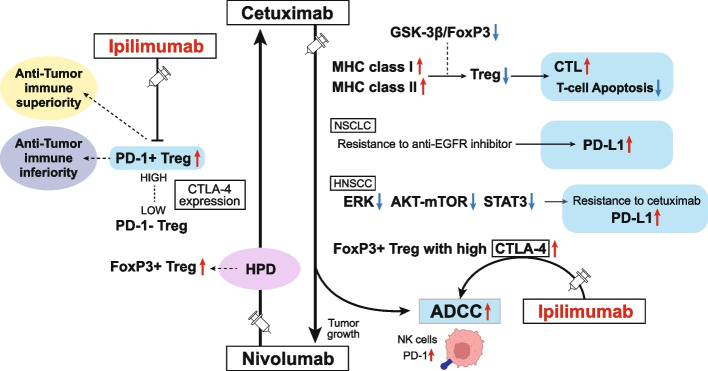
TME modulation by cetuximab and nivolumab and the potential breakthrough treatment strategy using ipilimumab for HPD. Inhibiting EGFR modulates the tumor immune microenvironment in several ways, including enhancing MHC class I and II expression, decreasing the suppressive function of Tregs, promoting CTL activity, and reducing T cell apoptosis. PD-1 blockade may facilitate the proliferation of highly suppressive PD-1 + effector Treg cells in HPDs, resulting in the inhibition of antitumor immunity. Highly activated tumor-infiltrating PD-1 + effector Treg cells show higher CTLA-4 expression levels than PD-1- effector Treg cells. As the PD-1 blockade significantly enhances Treg cell suppressive activity, PD-1 blockade facilitates the proliferation of highly suppressive PD-1 + effector Treg cells expressing high CTLA-4 in HPDs, resulting in immunosuppression in HNSCC. Moreover, cetuximab treatment increases the FoxP3 + intratumoral effector Tregs expressing CTLA-4, suggesting the combination with ipilimumab restores the cytolytic functions of NK cells mediating ADCC. Targeting CTLA-4 high PD-1 effector Tregs for HNSCC may show a high response to the tumor and improve survival

ICI treatment discontinuation is often due to tumor HPD, which cannot be controlled using ICIs (here, we do not consider the occurrence of severe immune-related adverse events as an exception). Thus, understanding the status of antitumor immunity in the HPD TME is important. Several studies on NSCLC have suggested that salvage chemotherapy after ICI treatment is highly effective in treating the disease [102, 103]. Furthermore, Kamada et al. reported the presence of highly proliferating FoxP3 + effector Tregs in patients with gastric cancer having HPD tumors after treatment with an anti-PD-1 antibody, in contrast to their absence in patients with non-HPD tumors. Functionally, highly activated circulating and tumor-infiltrating PD-1 + effector Treg cells show higher CTLA-4 expression levels than PD-1- effector Treg cells. As the PD-1 blockade significantly enhances Treg cell suppressive activity, PD-1 blockade may facilitate the proliferation of highly suppressive PD-1 + effector Treg cells expressing high CTLA-4 in HPDs, resulting in antitumor immunity inhibition. Thus, it could be considered that HPD occurs when PD-1 blockade activates and expands tumor-infiltrating PD-1 + effector Treg cells to overwhelm tumor-reactive PD-1 + effector T cells. Therefore, the presence of actively proliferating PD-1 + effector Treg cells in tumors is an indirect reliable marker for the progression to HPD, and targeting this may facilitate the treatment of HPD [104]. Actually, Matoba et al. discovered that Treg cells expressing abundant CTLA-4 on the cell surface were expanded in human HNSCC samples and suggested it as a new therapeutic target to evoke effective immune responses to HNSCC [105]. Moreover, Jie et al. reported that in HNSCC, cetuximab treatment increases FoxP3 + intratumoral effector Tregs expressing CTLA-4. Their investigation also revealed that ipilimumab (anti-CTLA-4 antibody) targeting CTLA-4 + Tregs restores the cytolytic functions of NK cells mediating ADCC. Treg-mediated immune suppression contributes to clinical response to cetuximab treatment was also investigated by them [4]. This observation suggests that its improvement by adding ipilimumab or via other Treg ablation strategies promotes antitumor immunity. Therefore, cetuximab treatment after anti-PD-1 treatment for R/M HNSCC, including rechallenge of cetuximab, might be modulated via combination treatment involving cetuximab and ipilimumab, may expect high response rates and improve survival for patients with HPD of HNSCC (Fig. 5). Thus, we hope the regimen of cetuximab plus ipilimumab is expected to be further examined basically and eventually proceed to clinical trials.

### Human papillomavirus (HPV) status and differences in biological behavior following ICI therapy

HNSCC has two main subtypes, namely, the HPV-related (HPV +) and HPV-unrelated (HPV −) subtypes. In general, HPV + HNSCC is more sensitive to treatment and shows better survival. In particular, the EXTREME trial showed that patients with p16 positivity benefit more from cetuximab therapy [106]. It has also been observed in patients in the Checkmate-141 subgroup with p16-positive tumors, the median overall survival was 9.1 months in the nivolumab group versus 4.4 months in the standard therapy group (hazard ratio for death, 0.56; 95% CI, 0.32 to 0.99); among patients with p16-negative tumors, the median overall survival was 7.5 versus 5.8 months (hazard ratio, 0.73; 95% CI, 0.42 to 1.25; *P* = 0.55 for interaction) [10]. This is because HPV infection alters the immune cell population infiltrating HNSCC, establishing a diverse and heterogeneous landscape with more immune infiltration. In addition, HPV-associated oncoproteins E5, E6, and E7 are key players in tumor cell metabolism. Specifically, E5 blocks HLA-C and HLA-E from the tumor stroma from interacting with MHC class I on cancer cells, thereby impairing T cell and NK cell activity [107]. Moreover, E5 attenuates MHC class II expression and stability by blocking peptide loading and transportation, and by interfering with MHC, it severely impairs antigen processing and T-cell activation [108]. Conversely, E6 and E7 proteins alter the NF-kB pathway in tumor cells, impair the innate immune system, and evade supervision [109]. They also interact with keratinocytes and inhibit macrophage infiltration [110]. Moreover, Luo et al. identified NLRX1 as a critical intermediary partner to facilitate HPV16 E7-potentiated STING turnover and the depletion of NLRX1 resulted in significantly improved IFN-I-dependent T-cell infiltration profiles and tumor control [111]. Other, EGFR amplification and abnormal PI3K/AKT/mTOR pathway activation are frequently observed in HPV + HNSCC (80–90% of cases) [112–114]. This also contributes to the enhancement of anti-EGFR therapy using cetuximab.

## Discussion

The biological background of cancer treatment remains unclear and reported trials in this regard lack promising background evidence. Moreover, it is expected that changes in the tumor immune microenvironment owing to the administration of cetuximab and nivolumab (or pembrolizumab) would influence the effects of the chemotherapeutic agents used on immunocompetent and immunosuppressive cells, with the said effects enhanced by interaction with the original anti-tumor effect if it is biologically compatible. In this review, we summarized the potential of modulating the TME after the administration of these targeted agents, discuss subsequent therapies, and suggest a reasonable combination therapy using cetuximab and an anti-CTLA-4 antibody that should be evaluated in the future as a breakthrough therapy for HPD of HNSCC including R/M diseases. However, further pre-clinical research is still needed to optimize the treatment sequence in HNSCC in order to maximize therapy options and to understand the impact of prior treatments on response to subsequent agents. Actually, the number of pre-clinical research models that are along with the aforementioned hypothesis is rare. This review objects primarily to presenting the hypotheses and perspectives obtained by combining the dispersed pieces of evidence. Given that the basic research on regimens involving the administration of an ICI followed by cetuximab treatment is limited, future research should include the evaluation of the biological efficacy of the regimen involving cetuximab administration following ICI to determine the detailed-biological efficacy of cetuximab, also including the cetuximab rechallenging. As our next concern, these targeted therapies need to be investigated given that they may promote the appearance of cancer stem cells in the TME as well as the circulation of these cells. This is also partly because, under such conditions, these cells may become resistant to the drugs.

## Data Availability

Not applicable.

## Abbreviations

ADCC: Antibody-dependent cellular cytotoxicity
ADCP: Antibody-dependent cellular phagocytosis
AIM2: Absent in melanoma 2
APC: Antigen-presenting cell
CRC: Colorectal cancer
CTL: Cytotoxic T-lymphocyte
CTLA-4: Cytotoxic T lymphocyte associated antigen
DAMP: Danger-associated molecular pattern
DC: Dendritic cells
EGFR: Epidermal growth factor receptor
HIF-1α: Hypoxia inducible factor-1 alpha
HNSCC: Head and neck squamous cell carcinoma
HPD: Hyper progression disease
HPV: Human papillomavirus
ICI: Immune checkpoint inhibitor
IFN-γ: Interferon-gamma
IL: Interleukin
JAK: Janus kinase
LAG-3: Lymphocyte-activation gene
MAPK: Mitogen-activated protein kinase
MDSC: Myeloid-derived suppressor cell
MEF: Mouse embryonic fibroblasts
mTOR: Mechanistic target of rapamycin
NK: Natural killer
NO: Nitric oxide
NOS2: Nitric oxide synthase 2
R/M: Recurrent or metastatic
PD-L1: Programmed cell death ligand 1
PD-1: Programmed cell death 1
STAT: Signal transducer and activator of transcription
STING: Stimulator of IFN genes
TAM: Tumor-associated macrophage
TCR: T cell receptor
TIM-3: T-cell immunoglobulin and mucin- domain containing
TME: Tumor microenvironment
Treg: Regulatory T cells
VEGF: Vascular endothelial growth factor
